# Long-term effect of thermoablative fractional CO_2_ laser treatment as a novel approach to urinary incontinence management in women with genitourinary syndrome of menopause

**DOI:** 10.1007/s00192-017-3352-1

**Published:** 2017-05-18

**Authors:** Pablo González Isaza, Kinga Jaguszewska, Jose Luis Cardona, Mariusz Lukaszuk

**Affiliations:** 1 Department of Urogynecology, San Jorge University Hospital, Pereira, Colombia; 2 Department of Obstetrics, Medical University of Gdansk, Kliniczna 1a, 80-402 Gdansk, Poland; 3 Medical Centre Nowe Orlowo, Gdynia, Poland; 4 Department of Pathology, Technological University of Pereira, Pereira, Colombia

**Keywords:** Stress urinary incontinence, Laser therapy, Menopause

## Abstract

**Introduction and hypothesis:**

The aim of this study was to evaluate the long-term effect of thermoablative fractional CO_2_ laser (TACO2L) as an alternative treatment for early stages of stress urinary incontinence (SUI) in postmenopausal women with genitourinary syndrome of menopause.

**Methods:**

A total of 161 postmenopausal patients (age 53.38 ± 5.1 years, range 45–65 years) with a clinical diagnosis of mild SUI were prospectively enrolled in the study. Patients received one treatment with TACO2L every 30–45 days, each treatment comprising four sessions, followed in all patients by a yearly treatment session at 12, 24 and 36 months. SUI was evaluated using the International Continence Society 1-h pad test and the International Consultation on Incontinence Questionnaire-Urinary Incontinence Short Form (ICIQ-UI SF) before and after TACO2L treatment.

**Results:**

TACO2L treatment was associated with a significant improvement in ICIQ-UI SF scores and 1-h pad weight test at 12 months (both *p* < 0.001), 24 months (both *p* < 0.001) and 36 months (both *p* < 0.001). Improvements were maintained for up to 36 months without the need for any further intervention. The results were confirmed by significant histological changes related to trophic restoration of the vagina, responsible for extrinsic and intrinsic mechanisms involved in urinary continence.

**Conclusions:**

Our results suggest that TACO2L is an efficient and safe novel treatment strategy in patients with mild SUI. Further investigation to confirm the long-term results presented here is still warranted.

## Introduction

Genitourinary syndrome of menopause (GSM), previously called vulvovaginal atrophy, manifests as morphological changes of the vaginal mucosa including thinning of the epithelium and loss of the vaginal folds, and reductions in blood flow and vaginal secretion, that alter the vaginal bacterial flora. GSM results in vaginal dryness, itching, burning, irritation, dysuria, dyspareunia, and irritative symptoms of the lower urinary tract including urinary frequency, urgency, incontinence and recurrent urinary tract infections [1, 2]. Urinary incontinence is related to pelvic floor dysfunction and is caused by relaxation of the anatomical structure that supports the periurethral tissue and impairment of the urethral sphincter, which is responsible for intrinsic and extrinsic continence. Stress urinary incontinence (SUI) remains the most common type of urinary incontinence [3] and is characterized by altered metabolism of the connective tissue that causes decreased collagen production leading to insufficient support of the urogenital tract [3–5]. Therefore, the medical use of thermal energy, which is already used in the fields of dermatology and esthetic medicine, was introduced as a potential strategy for the treatment of urinary incontinence. Laser energy may induce collagen denaturation, remodeling and neogenesis [6] giving immediate tightening of collagen fibril formations and more elastic tissue after treatment [4]. Transvaginal use of the thermoablative fractional CO_2_ laser (TACO2L) can activate collagen to promote elastin formation and modulate the activation of metalloproteinases at the molecular level.

The aim of this novel treatment strategy is to generate controlled thermal damage with a further wound healing response that physiologically stimulates fibroblasts to produce neocollagenesis [5, 7]. There is still a lack of understanding of the histological changes that promote biostimulation and restoration of the aging changes in the urogenital tract. Thus further research on the effectiveness of the thermal energy laser for the treatment of SUI is needed. The availability of such a nonsurgical approach would allow surgical intervention to be avoided or postponed. We present the first long-term results of the use of TACO2L in the treatment of mild SUI. We sought to demonstrate subjective and objective improvements in SUI associated with GSM. Additionally, we describe the histological changes that occur in trophic restoration of the vagina at the urethrovesical junction.

## Materials and methods

A total of 161 postmenopausal women (age range 45–65 years) with a diagnosis of mild SUI were prospectively enrolled at the Urogynecology Unit, San Jorge University Hospital, Pereira, Colombia, between September and December 2015. Patients with genital prolapse of Pelvic Organ Prolapse Quantification (POP-Q) stage >I in the anterior compartment and patients who were not adequately classified because of previous surgery, recurrent lower urinary tract infection or obesity (body mass index >35 kg/m^2^) were excluded from the study.

Written informed consent was obtained from all patients. The study was approved by the appropriate medical ethics committee and institutional review board.

Patients received laser treatment comprising four sessions every 30–45 days, followed by a protocol-based yearly treatment session at 12, 24 and 36 months performed with a SmartXide^2^ V^2^LR fractional microablative CO_2_ laser system (MonaLisa Touch™; Deka, Florence, Italy) at the urethrovesical junction. The same laser device parameters were used in all patients. Punch biopsies were obtained at the urethrovesical junction in the anterior compartment before and at the end of the treatment protocol. Sections of the blinded biopsy samples were stained with hematoxylin and eosin for histological analysis at baseline, and 6 weeks and 6 months after treatment of the vaginal walls with TACO2L, with emphasis at the level of at the urethrovesical junction (Gonzalez ironing technique). SUI was evaluated using the International Consultation on Incontinence Questionnaire-Urinary Incontinence Short Form (ICIQ-UI SF) and the International Continence Society 1-h pad test before and after each TACO2L treatment. All patients attended the follow-up visits at 12-, 24-, and 36 months.

## Statistical analysis

Statistical analyses were performed using SPSS Advanced Statistics version 22 (IBM Corp., Armonk, NY). Continuous variables were first checked for the normality of their distribution using the Shapiro-Wilk test. Mean values with standard deviations (SD) were derived from normally distributed parameters. Normally distributied variables were compared using Student’s *t* test. Categorical variables are expressed as counts and percentages and were compared using Fisher’s exact test. Standardized differences were calculated as means or proportion differences divided by the SD of the difference. The statistical tests were two-tailed, and a *p* value <0.05 was considered to indicate statistical significance.

## Results

**Fig. 1:**
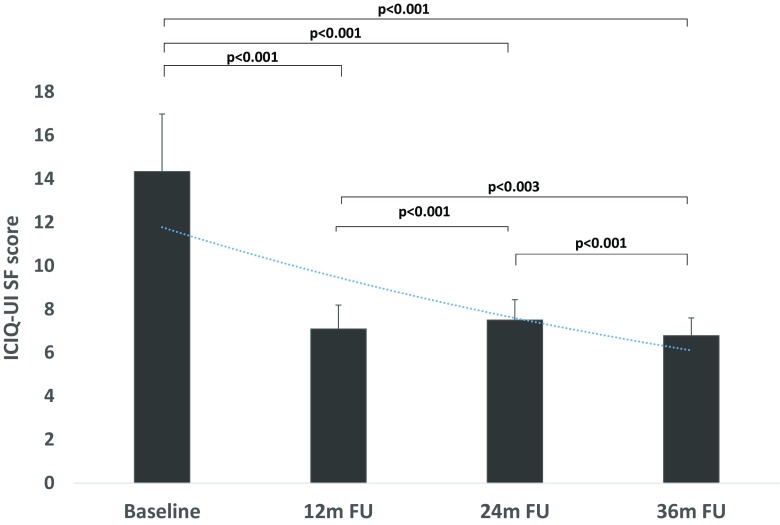
ICIQ-UI SF scores at baseline and at 12, 24 and 36 months after TACO2L, shown as mean values with standard deviations

**Fig. 2:**
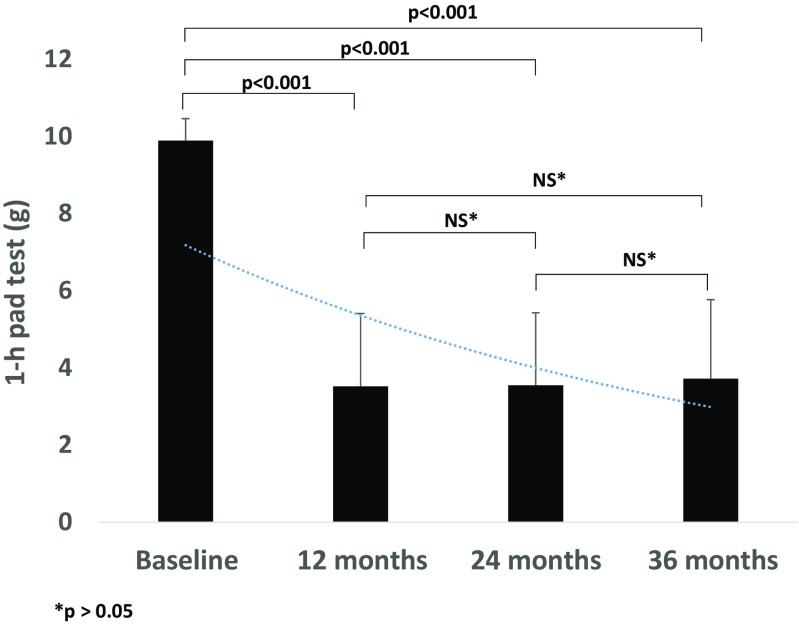
1-h pad test results at baseline and at 12, 24 and 36 months after TACO2L, shown as mean values with standard deviations

**Fig. 3:**
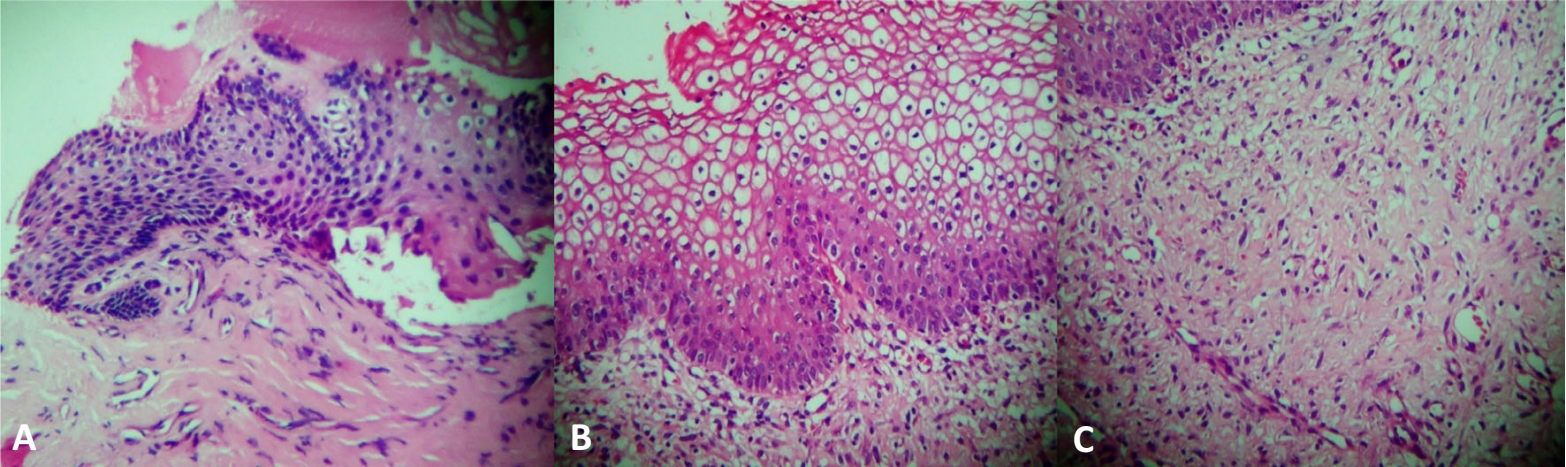
Sections of samples of vaginal mucosa stained with hematoxylin and eosin from a patient before treatment (**a**) and 6 weeks after treatment (**b**, **c**). **a** The epithelium of the atrophic mucosa is very thin, containing few layers of cells. **b** The epithelium appears much thicker with a higher population of intermediate and shedding superficial cells. The underlying connective tissue is much better organized both in the lamina propria and in the core of papille sible, with papillae indenting the epithelium-connective tissue junction. **c** The stroma shows features indicating structural recovery that was seen in all patients 6 weeks after treatment

Baseline characteristics of the study population are presented in Table 1. TACO2L treatment was well tolerated, and no side effects were observed during the study period. TACO2L treatment resulted in improvements in ICIQ-UI SF scores at 12 months (14.34 ± 2.65 vs. 7.09 ± 1.1, *p* < 0.001), 24 months (14.34 ± 2.65 vs. 7.49 ± 0.94, *p* < 0.001), and 36 months (14.34 ± 2.65 vs. 6.76 ± 0.82, *p* < 0.001; Fig. 1). Although there was a significant increase in ICIQ-UI SF score between 12 months and 24 months (7.09 ± 1.1 vs. 7.49 ± 0.94, *p* < 0.001), there was a significant decrease between 24 months and 36 months (*p* < 0.001) and overall between 12 months and 36 months (*p* < 0.003). At the end of the treatment protocol, 32% of patients (51) reported having moderate urinary incontinence. Furthermore, significant changes were recorded in the 1-h pad weight test from 9.89 ± 0.57 g at baseline to 3.52 ± 1.89 g, 3.55 ± 1.88 g, and 3.72 ± 2.05 g at 12, 24 and 36 months, respectively (all *p* < 0.001; Fig. 2). There were no differences in the 1-h pad weight test results between the follow-up time points 12 months, 24 months and 36 months (all *p* > 0.05; Fig. 2). Histological examination of the vaginal mucosa revealed essentially thicker epithelium with a higher population of intermediate and shedding superficial cells and underlying connective tissue with papillae indenting the epithelium–connective tissue junction. The stroma showed features indicating structural recovery in all patients at 6 weeks (Fig. 3).

**Table 1:** Baseline characteristics of the 161 patients included in the study

Characteristic	Value
Age (years), mean ± SD	53 ± 5.1
Parity, mean ± SD	2.5 ± 1.0
Menopause, *n* (%)	161 (100)
Hormone replacement therapy, *n* (%)	40 (36.6)
Sexually active, *n* (%)	140 (86.95)
POP-Q stage	
0	40 (25)
I	20 (32)

## Discussion

The present prospective study investigated for the first time the efficacy and safety of TACO2L a novel method for the treatment of patients with mild SUI. The most salient finding of the present study is that the treatment with TACO2L significantly improved ICIQ-UI SF scores and 1-h pad weight test results, and the improvements were maintained during the 36 months of observation without any need for further reintervention. The results were confirmed by crucial histological changes indicating trophic restoration of the vagina, that is responsible for important extrinsic and intrinsic mechanisms involved in urinary continence. These observations suggest that TACO2L is an effective and safe procedure for the treatment of mild SUI.

Currently, there are many initial nonsurgical therapies for patients with SUI, e.g. behavioral therapy, pelvic floor muscle exercises, electric stimulation, vaginal cones, occlusive and intravaginal devices, and pharmacological treatment. However, these strategies require the patient to show patience, motivation and time commitment, and to undergo training. Women are unlikely to comply with a strict program of behavior modification and regular pelvic floor exercises.

Thus, although surgery is more invasive and is burdensome in terms of complications and recovery time, it still remains a more attractive and useful option for the treatment of SUI. Therefore, other noninvasive methods are still urgently required.

Previous studies have shown molecular changes and collagen synthesis in the atrophic vagina after fractional CO_2_ treatment [5, 8]. The biostimulative effect of TACO2L restores most vaginal functions, including secretion, absorption, elasticity and lubrication, and the thickness of the vaginal epithelium [8]. It has been proposed that the production of elastic fibers and stimulation of neocollagenesis, that leads to increased thickness of the vaginal epithelium may be related to the restoration of urethral coaptation mechanisms involved in the physiopathology of SUI [8, 9]. Furthermore, recent pilot studies have shown remarkable improvement in SUI after laser energy treatment including recovery of the submucosal blood vessel plexus and an increase in periurethral muscle tone that is related to the urethral closure pressure mechanism [8, 9].

Radiofrequency ablation is used in the laparoscopic and transurethral treatment of SUI [10–14]. Fistonic et al. performed the pilot study in 39 patients with mild-to-moderate SUI with or without prolapse and revealed that nonablative fractional laser treatment with the IncontiLase™ system was efficient, safe and comfortable [4]. However, Fistonic et al. used only the ICIQ-UI SF and the Q-tip test to assess the efficacy of the method. Tien et al. demonstrated the feasibility of the IncontiLase system for the treatment of mild SUI with a follow-up of 6 months but found no effect in patients with a pad weight >10 g [14]. They found that treatment with the IncontiLase system improves lower urinary tract symptoms, quality of life and sexual function of both partners [14]. However, the short follow-up, the lack of a control group and the small study population limits the value of the study results.

TACO2L therapy seems to be a promising alternative for the treatment of SUI and other symptoms related to GSM. However, there may be safety concerns among the scientific community. Therefore, we focused on investigating a new device that works in different pulse shape modes with computer-controlled fractional emission. The fractional CO_2_ laser penetrates, and therefore interacts with, the vaginal epithelium to a depth of not more than 0.6 mm. Therefore thermal diffusion to structures around the vagina such as the rectum, bladder and peritoneal cavity is not possible. Our study demonstrated that an adequate amount of heat is delivered to the deep vaginal epithelium and urogenital structures to generate histologically confirmed structural changes that seem to be related to vaginal trophic intrinsic and extrinsic continence mechanisms of the urethra. This treatment strategy should not be used in patients with advanced stage pelvic organ prolapse which is managed by site-specific repair with or without extirpative surgery. Furthermore, moderate and severe urinary incontinence should be treated by placement of a midurethral sling, which remains the first-line surgical procedure with a long-term cure rate of 77–85% [15, 16].

Thus, TACO2L should not replace invasive procedures in advanced stage urinary incontinence if a first-line therapy is ineffective. In this study we showed for the first time the long-term efficacy of this novel treatment strategy that may have an impact on future guidelines for the management of SUI. Of note, the improvements were maintained for up to 36 months without the need for any further intervention. Although, there was no difference in the 1-h pad test results between the follow-up time points, a significant increase in ICIQ-UI SF score was documented after 12 months (Figs. 1 and 2). This result indicates that sequential TACO2L may be needed following the baseline procedure.

TACO2L seems to be an attractive alternative method and the best option for patients with GSM and mild SUI, who either do not have a surgical indication and/or have contraindications to surgery or do not wish to undergo an invasive procedure. The lack of significant adverse effects, its noninvasive nature and significant improvement in ICIQ-UI SF scores and pad weight test results over a long follow-up, supported by histological analysis, confirm TACO2L as a beneficial treatment strategy that allows the need surgery to be avoided or postponed.

### Limitations of the study

This was a prospective, nonrandomized study of an observational nature with no control group. Since all patients enrolled were relatively young (45–65 years), the results of this particular study cannot be translated into older populations.

### Conclusion

Our results suggest that TACO2L is an efficient and safe novel strategy for the treatment of mild SUI. The TACO2L procedure should not only be applied in the field of esthetic gynecology as its primary goal is to restore intrinsic and extrinsic continence mechanisms related to the trophism of the vaginal epithelium and to improve long-term urinary incontinence related to GSM. Further investigation to confirm the long-term effects of TACO2L is still warranted.
